# (5R*)-5-[(2S*,5S*)-1-Meth­oxy-5-phenyl­pyrrolidin-2-yl]-3-methyl­furan-2(5*H*)-one

**DOI:** 10.1107/S1600536814014974

**Published:** 2014-07-02

**Authors:** Takeshi Oishi, Makoto Yoritate, Takaaki Sato, Noritaka Chida

**Affiliations:** a School of Medicine, Keio University, Hiyoshi 4-1-1, Kohoku-ku, Yokohama 223-8521, Japan; b Department of Applied Chemistry, Faculty of Science and Technology, Keio University, Hiyoshi 3-14-1, Kohoku-ku, Yokohama 223-8522, Japan

**Keywords:** crystal structure

## Abstract

In the title compound, C_16_H_19_NO_3_, the pyrrolidine ring is in a twist conformation. The dihedral angle between the di­hydro­furan ring [maximum deviation = 0.0016 (11) Å] and the phenyl ring is 47.22 (8)°. In the crystal, mol­ecules are linked by weak C—H⋯O hydrogen bonds, forming helical chains along the *b-*axis direction. The chains are further linked by C—H⋯π inter­actions to constitute a three-dimensional architecture.

## Related literature   

For noteworthy mild reactions of *N*-alk­oxy­amines, see: Hawker *et al.* (2001 ▶). For the reaction of Weinreb amide, see: Nahm & Weinreb (1981 ▶). For the synthesis of the title compound, see: Yoritate *et al.* (2014 ▶). For a related article utilizing similar compounds, see: Yanagita *et al.* (2013 ▶). For details of ring conformations, see: Cremer & Pople (1975 ▶).
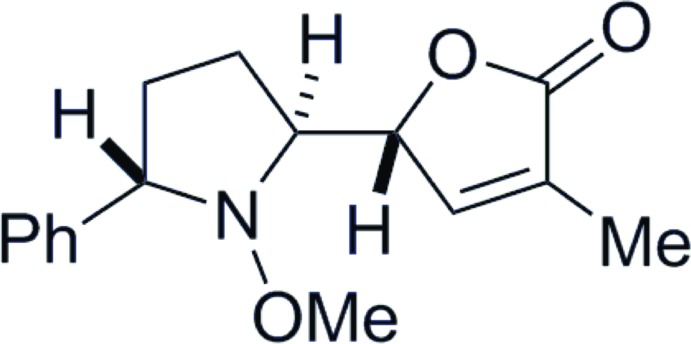


## Experimental   

### 

#### Crystal data  

C_16_H_19_NO_3_
*M* *_r_* = 273.32
Orthorhombic, 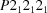

*a* = 6.5427 (3) Å
*b* = 10.8219 (5) Å
*c* = 19.8397 (10) Å
*V* = 1404.74 (12) Å^3^
*Z* = 4
Mo *K*α radiation
μ = 0.09 mm^−1^
*T* = 90 K
0.54 × 0.51 × 0.40 mm

#### Data collection  

Bruker D8 diffractometer
Absorption correction: multi-scan (*SADABS*; Bruker, 2012 ▶) *T*_min_ = 0.95, *T*_max_ = 0.97
12710 measured reflections
1510 independent reflections
1474 reflections with *I* > 2σ(*I*)
*R*_int_ = 0.027

#### Refinement  

*R*[*F* ^2^ > 2σ(*F* ^2^)] = 0.031
*wR*(*F* ^2^) = 0.075
*S* = 1.04
1510 reflections
184 parameters
H-atom parameters constrained
Δρ_max_ = 0.21 e Å^−3^
Δρ_min_ = −0.17 e Å^−3^

### 

Data collection: *APEX2* (Bruker, 2012 ▶); cell refinement: *SAINT* (Bruker, 2012 ▶); data reduction: *SAINT*; program(s) used to solve structure: *SHELXS97* (Sheldrick, 2008 ▶); program(s) used to refine structure: *SHELXL97* (Sheldrick, 2008 ▶); molecular graphics: *Mercury* (Macrae *et al.*, 2006 ▶); software used to prepare material for publication: *publCIF* (Westrip, 2010 ▶) and *PLATON* (Spek, 2009 ▶).

## Supplementary Material

Crystal structure: contains datablock(s) global, I. DOI: 10.1107/S1600536814014974/is5367sup1.cif

Structure factors: contains datablock(s) I. DOI: 10.1107/S1600536814014974/is5367Isup2.hkl

Supporting information file. DOI: 10.1107/S1600536814014974/is5367Isup3.cml

CCDC reference: 1010196

Additional supporting information:  crystallographic information; 3D view; checkCIF report

## Figures and Tables

**Table 1:** Hydrogen-bond geometry (Å, °) *Cg*1 and *Cg*3 are the centroids of the O1/C2–C5 di­hydro­furan and C15–C20 phenyl rings, respectively.

*D*—H⋯*A*	*D*—H	H⋯*A*	*D*⋯*A*	*D*—H⋯*A*
C5—H5⋯O6^i^	1.00	2.51	3.185 (2)	125
C10—H10*A*⋯*Cg*1^ii^	0.99	2.89	3.686 (2)	138
C16—H16⋯*Cg*3^iii^	0.95	2.99	3.761 (2)	139

Symmetry codes: (i) 
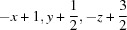
; (ii) 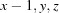; (iii) 
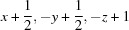
.

**Fig. 1.**
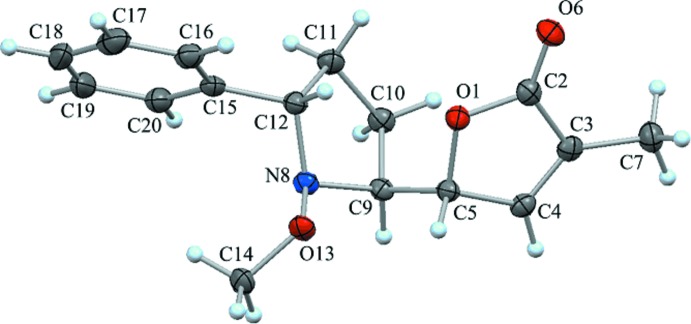
The molecular structure of the title compound, with atom labels and 50% probability displacement ellipsoids for non-H atoms.

**Fig. 2.**
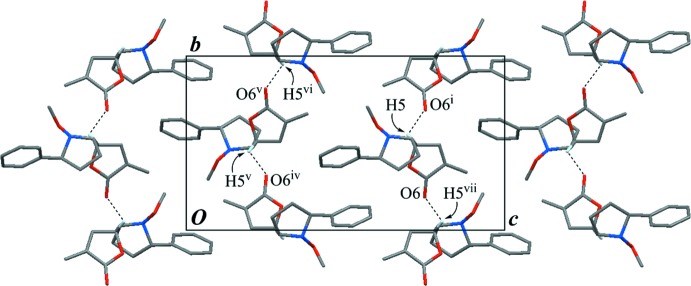
The crystal packing of the title compound, viewed down the a axis. Dashed lines indicate the intermolecular C5—H···O6 interactions, making helical chains along [010]. Only H atoms involved in hydrogen bonds were shown for clarity. Symmetry codes: (i) –x + 1, y + 1/2, –z + 3/2; (iv) x – 1/2, –y + 1/2, –z + 1; (v) –x + 1/2, –y + 1, z – 1/2; (vi) x – 1/2, –y + 3/2, –z + 1; (vii) –x + 1, y – 1/2, –z + 3/2.

**Fig. 3.**
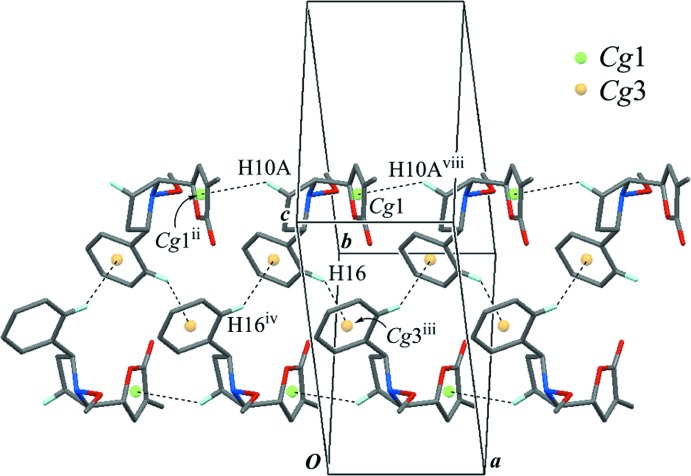
A view for the intermolecular C—H···π interactions (dashed lines), showing parallel (C10—H10A···Cg1) and alternated (C16—H16···Cg3) chains along [100]. Cg1 and Cg3 are the centroids of the O1/C2–C5 dihydrofuran and the C15–C20 phenyl rings, respectively. Only H atoms involved in hydrogen bonds were shown for clarity. Symmetry codes: (ii) x – 1, y, z; (iii) x + 1/2, –y + 1/2, –z + 1; (iv) x – 1/2, –y + 1/2, –z + 1; (viii) x + 1, y, z.

**Fig. 4.**
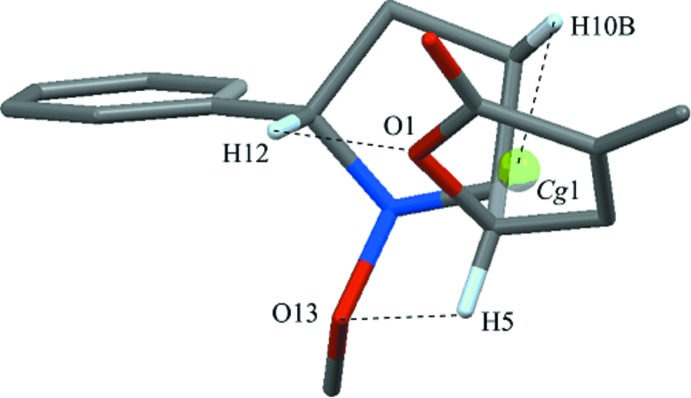
Molecular conformation indicating intramolecular C—H···O and C—H···π interactions with dashed lines. Cg1 is a centroid of the O1/C2–C5 dihydrofuran ring.

## supplementary crystallographic information

### S1. Comment

A number of compounds containing oxidized nitrogen functionality have been widely used in organic synthesis. In these substances, the *N*-alkoxyamines are known as the initiators for the stable free radical polymerization (Hawker *et al.*, 2001), and the *N*-alkoxyamides are utilized for mild and effective acylating agents (*cf.* Weinreb amide; Nahm & Weinreb, 1981). We noticed this inert N—O covalent bond, to develop a novel reaction to synthesize the natural alkaloids (Yanagita *et al.*, 2013).

In the title compound, the dihydrofuran ring is planar with a maximum deviation of 0.0016 (11) Å at atom C4, and the pyrrolidine ring is in a twist conformation with puckering parameters of Q(2) = 0.4145 (18) Å and φ(2) = 10.6 (3)° (Cremer & Pople, 1975). Atoms N8 and C9 are deviated by –0.4566 (13) and 0.1991 (19) Å, respectively, from the plane of other carbon atoms (C10–C12). Angles of O13—N8—C9, O13—N8—C12 and C9—N8—C12 being 110.28 (13), 108.44 (12) and 106.87 (13)°, respectively, revealed the *sp*^3^ configuration of the N8 atom. The relative configurations were confirmed by the X-ray analysis as C5*R*, C9*S* and C12*S*.

The crystal packing iss stabilized by an intermolecular C5—H5···O6 (–*x* + 1, *y* + 1/2, –*z* + 3/2) hydrogen bond (Table 1), forming a helical chain along to the [010] direction (Fig. 2). Further intermolecular C—H···π interactions form a three-dimensional network in the crystal structure (Fig. 3). Distances for C10—H10A···*Cg*1 (*x* – 1, *y*, *z*) and C16—H···*Cg*3 (*x* + 1/2, –*y* + 1/2, –*z* + 1) are 3.686 (2) and 3.761 (2) Å, respectively. *Cg*1 and *Cg*3 are the centroids of the O1/C2–C5 dihydrofuran and C15–C20 phenyl rings, respectively. Additionally, weak intramolecular interactions, C12—H···O1, C5—H···O13 and C10—H10B···*Cg*1 being 2.957 (2), 2.791 (2) and 2.963 (2) Å, respectively, adopt the molecule into a sterically hindered conformation. The C5—O1 bond of dihydrofuran is overhanged on the pyrrolidine ring, with torsion angles of O1—C5—C9—N8 and O1—C5—C9—C10 being –69.7 (2) and 46.5 (2)°, respectively (Fig. 4).

### S2. Experimental

The title compound was synthesized from 4-oxo-4-phenylbutyric acid (Yoritate *et al.*, 2014), and recrystallized from a toluene solution by slow evaporation at ambient temperature; M.p. 358.5–359.9 K (not corrected). ^1^H NMR (500 MHz, CDCl_3_) *δ* (p.p.m.) = 7.41–7.37 (m, 2H, Ph), 7.36–7.31 (m, 2H, Ph), 7.29–7.24 (m, 1H, Ph), 7.13 (qd, *J* = 1.7, 1.7 Hz, 1H, H4), 5.35–5.31 (m, 1H, H5), 4.33 (dd, *J* = 8.2, 7.5 Hz, 1H, H12), 3.56 (ddd, *J* = 8.3, 4.9, 4.9 Hz, 1H, H9), 3.35 (s, 3H, OMe), 2.20 (dddd, *J* = 12.9, 10.0, 7.5, 4.0 Hz, 1H, H11A), 2.00 (dddd, *J* = 13.1, 10.3, 8.3, 4.0 Hz, 1H, H10A), 1.95 (dd, *J* = 1.7, 1.7 Hz, 3H, CMe), 1.93–1.84 (m, 1H, H11B), 1.62 (dddd, *J* = 13.1, 10.0, 6.6, 4.9 Hz, 1H, H10B); ^13^C NMR (125 MHz, CDCl_3_) *δ* (p.p.m.) = 174.6 (C), 148.1 (CH), 141.1 (C), 130.7 (C), 128.3 (CH), 128.1 (CH), 127.4 (CH), 80.5 (CH), 68.6 (CH), 65.3 (CH), 61.2 (CH_2_), 28.9 (CH_2_), 22.6 (CH_2_), 10.9 (CH_3_); Anal. calcd. for C_16_H_19_NO_3_: C 70.31, H 7.01, N 5.12%, found: C 70.15, H 7.00, N 5.06%.

### S3. Refinement

C-bound H atoms were positioned geometrically with C—H = 0.95–1.00 Å, and constrained to ride on their parent atoms with *U*_iso_(H) = 1.2*U*_eq_(C) or 1.5*U*_eq_(methyl C). The Friedel opposites were merged before the final refinement because no significant anomalous dispersion was observed and the Flack parameter was a meaningless value of –1.2 (10) with 1054 Bijvoet pairs. One reflection (7 3 4) has been omitted in the final refinement.

### Crystal data

**Table:**

C_16_H_19_NO_3_	*D*_x_ = 1.292 Mg m^−^^3^
*M**_r_* = 273.32	Melting point: 358.5 K
Orthorhombic, *P*2_1_2_1_2_1_	Mo *K*α radiation, λ = 0.71073 Å
*a* = 6.5427 (3) Å	Cell parameters from 9968 reflections
*b* = 10.8219 (5) Å	θ = 2.8–25.4°
*c* = 19.8397 (10) Å	µ = 0.09 mm^−^^1^
*V* = 1404.74 (12) Å^3^	*T* = 90 K
*Z* = 4	Prism, colourless
*F*(000) = 584	0.54 × 0.51 × 0.40 mm

### Data collection

**Table:**

Bruker D8 diffractometer	1510 independent reflections
Radiation source: fine-focus sealed tube	1474 reflections with *I* > 2σ(*I*)
Multilayered confocal mirror monochromator	*R*_int_ = 0.027
Detector resolution: 8.333 pixels mm^-1^	θ_max_ = 25.4°, θ_min_ = 2.8°
ω scans	*h* = −7→7
Absorption correction: multi-scan (*SADABS*; Bruker, 2012)	*k* = −13→11
*T*_min_ = 0.95, *T*_max_ = 0.97	*l* = −23→22
12710 measured reflections	

### Refinement

**Table:**

Refinement on *F*^2^	Secondary atom site location: difference Fourier map
Least-squares matrix: full	Hydrogen site location: inferred from neighbouring sites
*R*[*F*^2^ > 2σ(*F*^2^)] = 0.031	H-atom parameters constrained
*wR*(*F*^2^) = 0.075	*w* = 1/[σ^2^(*F*_o_^2^) + (0.0428*P*)^2^ + 0.402*P*] where *P* = (*F*_o_^2^ + 2*F*_c_^2^)/3
*S* = 1.04	(Δ/σ)_max_ = 0.014
1510 reflections	Δρ_max_ = 0.21 e Å^−^^3^
184 parameters	Δρ_min_ = −0.17 e Å^−^^3^
0 restraints	Extinction correction: *SHELXL*
Primary atom site location: structure-invariant direct methods	Extinction coefficient: 0.029 (3)

### Special details

**Table:**

Geometry. All e.s.d.'s (except the e.s.d. in the dihedral angle between two l.s. planes) are estimated using the full covariance matrix. The cell e.s.d.'s are taken into account individually in the estimation of e.s.d.'s in distances, angles and torsion angles; correlations between e.s.d.'s in cell parameters are only used when they are defined by crystal symmetry. An approximate (isotropic) treatment of cell e.s.d.'s is used for estimating e.s.d.'s involving l.s. planes.
Refinement. Refinement of *F*^2^ against ALL reflections. The weighted *R*-factor *wR* and goodness of fit *S* are based on *F*^2^, conventional *R*-factors *R* are based on *F*, with *F* set to zero for negative *F*^2^. The threshold expression of *F*^2^ > σ(*F*^2^) is used only for calculating *R*-factors(gt) *etc*. and is not relevant to the choice of reflections for refinement. *R*-factors based on *F*^2^ are statistically about twice as large as those based on *F*, and *R*- factors based on ALL data will be even larger.

### Fractional atomic coordinates and isotropic or equivalent isotropic displacement parameters (Å^2^)

**Table:**

	*x*	*y*	*z*	*U*_iso_*/*U*_eq_	
O1	0.28934 (19)	0.36404 (11)	0.70066 (5)	0.0197 (3)	
C2	0.3603 (3)	0.29777 (16)	0.75407 (8)	0.0200 (4)	
C3	0.3704 (3)	0.37890 (16)	0.81354 (8)	0.0198 (4)	
C4	0.3059 (3)	0.48928 (16)	0.79475 (8)	0.0198 (4)	
H4	0.2965	0.559	0.8237	0.024*	
C5	0.2496 (3)	0.48950 (17)	0.72169 (8)	0.0181 (4)	
H5	0.3424	0.5469	0.6967	0.022*	
O6	0.4060 (2)	0.19033 (12)	0.74867 (6)	0.0268 (3)	
C7	0.4522 (3)	0.33239 (18)	0.87893 (8)	0.0272 (4)	
H7A	0.4346	0.3959	0.9137	0.041*	
H7B	0.5978	0.3132	0.874	0.041*	
H7C	0.3779	0.2576	0.8921	0.041*	
N8	−0.0284 (2)	0.53897 (13)	0.63598 (7)	0.0185 (3)	
C9	0.0267 (3)	0.52517 (16)	0.70776 (8)	0.0179 (4)	
H9	−0.0038	0.6045	0.7316	0.021*	
C10	−0.1287 (3)	0.42769 (16)	0.73027 (8)	0.0209 (4)	
H10A	−0.2569	0.4672	0.7457	0.025*	
H10B	−0.0728	0.3768	0.7674	0.025*	
C11	−0.1671 (3)	0.34798 (17)	0.66697 (8)	0.0245 (4)	
H11A	−0.1119	0.2636	0.6735	0.029*	
H11B	−0.3154	0.3418	0.6576	0.029*	
C12	−0.0570 (3)	0.41297 (15)	0.60873 (8)	0.0193 (4)	
H12	0.0794	0.3736	0.6014	0.023*	
O13	0.13547 (19)	0.59737 (11)	0.59960 (6)	0.0208 (3)	
C14	0.0574 (3)	0.70653 (17)	0.56898 (9)	0.0251 (4)	
H14A	−0.0573	0.6851	0.5394	0.038*	
H14B	0.1653	0.7461	0.5424	0.038*	
H14C	0.0103	0.7636	0.604	0.038*	
C15	−0.1725 (3)	0.41689 (16)	0.54286 (8)	0.0205 (4)	
C16	−0.0888 (3)	0.36521 (18)	0.48516 (8)	0.0258 (4)	
H16	0.0411	0.3262	0.4873	0.031*	
C17	−0.1934 (4)	0.36997 (19)	0.42435 (9)	0.0358 (5)	
H17	−0.1347	0.3345	0.385	0.043*	
C18	−0.3824 (4)	0.42609 (19)	0.42089 (10)	0.0398 (6)	
H18	−0.4529	0.4303	0.3791	0.048*	
C19	−0.4695 (4)	0.47615 (19)	0.47816 (11)	0.0370 (5)	
H19	−0.6006	0.5136	0.4758	0.044*	
C20	−0.3655 (3)	0.47190 (18)	0.53920 (10)	0.0276 (4)	
H20	−0.4257	0.5064	0.5785	0.033*	

### Atomic displacement parameters (Å^2^)

**Table:**

	*U*^11^	*U*^22^	*U*^33^	*U*^12^	*U*^13^	*U*^23^
O1	0.0201 (6)	0.0197 (6)	0.0194 (5)	0.0030 (5)	−0.0008 (5)	−0.0022 (5)
C2	0.0136 (8)	0.0222 (9)	0.0241 (8)	0.0007 (7)	0.0023 (7)	0.0019 (7)
C3	0.0147 (8)	0.0230 (8)	0.0218 (8)	−0.0029 (8)	0.0017 (7)	0.0009 (7)
C4	0.0176 (9)	0.0211 (8)	0.0208 (8)	−0.0024 (8)	0.0002 (7)	−0.0018 (7)
C5	0.0195 (9)	0.0159 (8)	0.0188 (8)	−0.0004 (7)	0.0000 (7)	0.0002 (6)
O6	0.0274 (7)	0.0209 (6)	0.0321 (6)	0.0070 (6)	−0.0013 (6)	−0.0006 (5)
C7	0.0262 (10)	0.0310 (10)	0.0243 (8)	0.0009 (9)	−0.0032 (8)	0.0054 (8)
N8	0.0179 (7)	0.0181 (7)	0.0194 (7)	−0.0025 (6)	0.0022 (6)	0.0024 (6)
C9	0.0182 (9)	0.0168 (8)	0.0187 (8)	0.0017 (7)	0.0014 (7)	0.0000 (7)
C10	0.0175 (8)	0.0214 (9)	0.0237 (8)	−0.0005 (8)	0.0027 (7)	0.0025 (7)
C11	0.0253 (10)	0.0231 (9)	0.0251 (8)	−0.0063 (8)	−0.0019 (8)	0.0039 (7)
C12	0.0198 (9)	0.0159 (8)	0.0223 (8)	0.0000 (7)	−0.0003 (7)	0.0002 (7)
O13	0.0178 (6)	0.0212 (6)	0.0235 (6)	−0.0030 (5)	0.0019 (5)	0.0052 (5)
C14	0.0280 (10)	0.0247 (9)	0.0227 (8)	−0.0052 (8)	−0.0044 (8)	0.0078 (7)
C15	0.0238 (9)	0.0151 (8)	0.0226 (8)	−0.0051 (8)	−0.0028 (7)	0.0015 (6)
C16	0.0297 (10)	0.0222 (9)	0.0254 (8)	−0.0071 (9)	0.0004 (8)	0.0003 (7)
C17	0.0543 (14)	0.0300 (10)	0.0231 (8)	−0.0180 (12)	−0.0020 (9)	0.0010 (8)
C18	0.0557 (15)	0.0305 (11)	0.0330 (10)	−0.0178 (11)	−0.0238 (11)	0.0103 (9)
C19	0.0349 (12)	0.0220 (10)	0.0541 (13)	−0.0045 (9)	−0.0221 (11)	0.0069 (9)
C20	0.0268 (10)	0.0198 (9)	0.0362 (10)	−0.0017 (9)	−0.0062 (9)	−0.0002 (8)

### Geometric parameters (Å, º)

**Table:**

O1—C2	1.361 (2)	C11—C12	1.532 (2)
O1—C5	1.444 (2)	C11—H11A	0.99
C2—O6	1.205 (2)	C11—H11B	0.99
C2—C3	1.472 (2)	C12—C15	1.510 (2)
C3—C4	1.321 (3)	C12—H12	1.0
C3—C7	1.491 (2)	O13—C14	1.423 (2)
C4—C5	1.495 (2)	C14—H14A	0.98
C4—H4	0.95	C14—H14B	0.98
C5—C9	1.534 (3)	C14—H14C	0.98
C5—H5	1.0	C15—C16	1.387 (2)
C7—H7A	0.98	C15—C20	1.398 (3)
C7—H7B	0.98	C16—C17	1.388 (3)
C7—H7C	0.98	C16—H16	0.95
N8—O13	1.4385 (19)	C17—C18	1.380 (4)
N8—C9	1.477 (2)	C17—H17	0.95
N8—C12	1.479 (2)	C18—C19	1.382 (3)
C9—C10	1.532 (2)	C18—H18	0.95
C9—H9	1.0	C19—C20	1.390 (3)
C10—C11	1.544 (2)	C19—H19	0.95
C10—H10A	0.99	C20—H20	0.95
C10—H10B	0.99		
			
C2—O1—C5	109.38 (12)	C12—C11—C10	106.28 (14)
O6—C2—O1	121.57 (16)	C12—C11—H11A	110.5
O6—C2—C3	129.46 (17)	C10—C11—H11A	110.5
O1—C2—C3	108.97 (14)	C12—C11—H11B	110.5
C4—C3—C2	107.40 (14)	C10—C11—H11B	110.5
C4—C3—C7	131.73 (16)	H11A—C11—H11B	108.7
C2—C3—C7	120.80 (16)	N8—C12—C15	110.73 (13)
C3—C4—C5	110.71 (15)	N8—C12—C11	101.97 (13)
C3—C4—H4	124.6	C15—C12—C11	115.48 (15)
C5—C4—H4	124.6	N8—C12—H12	109.5
O1—C5—C4	103.55 (14)	C15—C12—H12	109.5
O1—C5—C9	110.83 (14)	C11—C12—H12	109.5
C4—C5—C9	114.14 (15)	C14—O13—N8	108.14 (13)
O1—C5—H5	109.4	O13—C14—H14A	109.5
C4—C5—H5	109.4	O13—C14—H14B	109.5
C9—C5—H5	109.4	H14A—C14—H14B	109.5
C3—C7—H7A	109.5	O13—C14—H14C	109.5
C3—C7—H7B	109.5	H14A—C14—H14C	109.5
H7A—C7—H7B	109.5	H14B—C14—H14C	109.5
C3—C7—H7C	109.5	C16—C15—C20	119.04 (17)
H7A—C7—H7C	109.5	C16—C15—C12	120.38 (17)
H7B—C7—H7C	109.5	C20—C15—C12	120.58 (16)
O13—N8—C9	110.28 (13)	C15—C16—C17	120.55 (19)
O13—N8—C12	108.44 (12)	C15—C16—H16	119.7
C9—N8—C12	106.87 (13)	C17—C16—H16	119.7
N8—C9—C10	100.89 (14)	C18—C17—C16	120.1 (2)
N8—C9—C5	115.55 (14)	C18—C17—H17	120.0
C10—C9—C5	113.92 (14)	C16—C17—H17	120.0
N8—C9—H9	108.7	C17—C18—C19	120.08 (19)
C10—C9—H9	108.7	C17—C18—H18	120.0
C5—C9—H9	108.7	C19—C18—H18	120.0
C9—C10—C11	104.81 (13)	C18—C19—C20	120.1 (2)
C9—C10—H10A	110.8	C18—C19—H19	119.9
C11—C10—H10A	110.8	C20—C19—H19	119.9
C9—C10—H10B	110.8	C19—C20—C15	120.10 (19)
C11—C10—H10B	110.8	C19—C20—H20	120.0
H10A—C10—H10B	108.9	C15—C20—H20	120.0
			
C5—O1—C2—O6	−179.35 (17)	C9—C10—C11—C12	−7.74 (19)
C5—O1—C2—C3	−0.06 (19)	O13—N8—C12—C15	−77.91 (17)
O6—C2—C3—C4	179.44 (19)	C9—N8—C12—C15	163.22 (14)
O1—C2—C3—C4	0.2 (2)	O13—N8—C12—C11	158.69 (13)
O6—C2—C3—C7	2.1 (3)	C9—N8—C12—C11	39.81 (18)
O1—C2—C3—C7	−177.16 (15)	C10—C11—C12—N8	−18.38 (18)
C2—C3—C4—C5	−0.3 (2)	C10—C11—C12—C15	−138.50 (15)
C7—C3—C4—C5	176.70 (18)	C9—N8—O13—C14	−122.23 (14)
C2—O1—C5—C4	−0.11 (18)	C12—N8—O13—C14	121.07 (14)
C2—O1—C5—C9	−122.92 (14)	N8—C12—C15—C16	123.43 (18)
C3—C4—C5—O1	0.25 (19)	C11—C12—C15—C16	−121.35 (18)
C3—C4—C5—C9	120.85 (17)	N8—C12—C15—C20	−57.0 (2)
O13—N8—C9—C10	−162.41 (13)	C11—C12—C15—C20	58.3 (2)
C12—N8—C9—C10	−44.74 (17)	C20—C15—C16—C17	1.2 (3)
O13—N8—C9—C5	−39.07 (19)	C12—C15—C16—C17	−179.20 (17)
C12—N8—C9—C5	78.60 (18)	C15—C16—C17—C18	−0.2 (3)
O1—C5—C9—N8	−69.67 (17)	C16—C17—C18—C19	−0.9 (3)
C4—C5—C9—N8	173.89 (15)	C17—C18—C19—C20	1.0 (3)
O1—C5—C9—C10	46.50 (18)	C18—C19—C20—C15	0.0 (3)
C4—C5—C9—C10	−69.9 (2)	C16—C15—C20—C19	−1.1 (3)
N8—C9—C10—C11	30.75 (17)	C12—C15—C20—C19	179.31 (17)
C5—C9—C10—C11	−93.70 (17)		

### Hydrogen-bond geometry (Å, º)

*Cg*1 and *Cg*3 are the centroids of the O1/C2–C5 dihydrofuran and C15–C20 phenyl rings, respectively.

**Table:**

*D*—H···*A*	*D*—H	H···*A*	*D*···*A*	*D*—H···*A*
C12—H12···O1	1.00	2.40	2.957 (2)	114
C5—H5···O13	1.00	2.42	2.791 (2)	101
C10—H10*B*···*Cg*1	0.99	2.56	2.963 (2)	104
C5—H5···O6^i^	1.00	2.51	3.185 (2)	125
C10—H10*A*···*Cg*1^ii^	0.99	2.89	3.686 (2)	138
C16—H16···*Cg*3^iii^	0.95	2.99	3.761 (2)	139

Symmetry codes: (i) −*x*+1, *y*+1/2, −*z*+3/2; (ii) *x*−1, *y*, *z*; (iii) *x*+1/2, −*y*+1/2, −*z*+1.
